# The attribution of success when using navigation aids

**DOI:** 10.1080/00140139.2014.977827

**Published:** 2014-11-11

**Authors:** Michael Brown, Robert Houghton, Sarah Sharples, Jeremy Morley

**Affiliations:** ^a^ Horizon Digital Economy Research, University of Nottingham, C01, Nottingham Geospatial Building, NottinghamNG7 2TU, UK; ^b^ Human Factors Research Group, University of Nottingham, Nottingham, UK; ^c^ Nottingham Geospatial Institute, University of Nottingham, Nottingham, UK

**Keywords:** personal navigation, attribution, trust in automation, human factors, attitudes and behaviour

## Abstract

Attitudes towards geographic information technology is a seldom explored research area that can be explained with reference to established theories of attribution. This article reports on a study of how the attribution of success and failure in pedestrian navigation varies with level of automation, degree of success and locus of control. A total of 113 participants took part in a survey exploring reflections on personal experiences and vignettes describing fictional navigation experiences. A complex relationship was discovered in which success tends to be attributed to skill and failure to the navigation aid when participants describe their own experiences. A reversed pattern of results was found when discussing the navigation of others. It was also found that navigation success and failure are associated with personal skill to a greater extent when using paper maps, as compared with web-based routing engines or satellite navigation systems.

**Practitioner Summary**: This article explores the influences on the attribution of success and failure when using navigation aids. A survey was performed exploring interpretations of navigation experiences. Level of success, self or other as navigator and type of navigation aid used are all found to influence the attribution of outcomes to internal or external factors.

## 1. Introduction

As the field of geographic information (GI) science advances, researchers are starting to look beyond the technical challenges in this domain to the human factors that influence the use of GI technologies (Sharples et al. 2013). The majority of work in this area to date has focused on issues surrounding usability, such as the design of usable navigation interfaces (Delikostidis et al. 2013) and GI usability (Brown et al. 2013). In this article, we explore attitudes towards navigation aids, specifically the attribution of success and failure when these aids are used for pedestrian navigation.

Every few months the media reports another story about drivers being duped by their satellite navigation system into performing dangerous and illegal manoeuvres on the roads. For example, Simpson (2008) reports that over 300,000 accidents have been caused in Britain by ‘Sat Nav Blunders’. These stories inevitably attract both technophobes damning our reliance on navigation technologies and technophiles placing all the blame on the driver. The truth of the matter varies from case to case, but may often lie somewhere between the two (Svenson, Lekberg, and Johansson 1999).

Anecdotally, these stories tend to focus only on satellite navigation systems used in vehicles, never mentioning accidents that may have happened due to the use of traditional paper maps or even web-based routing engines. In order to understand the underlying features that influence attitudes towards the use of technologically mediated aids to support navigation, this article explores how users attribute navigation success and failure when using navigation aids, be they paper maps, satellite navigation or web-based routing engines. We also discuss the methodological implications of reporting on personal experiences and describing the behaviour of others.

### 1.1 Attribution and bias

Attribution theory suggests two core effects that might be relevant in this domain. On the one hand, self-serving bias describes the tendency to attribute personal success to the self and failure to external factors (Miller and Ross 1975). On the other hand, fundamental attribution error is the well-documented ‘tendency to overestimate dispositional and underestimate situational causes of others' behaviour’ (Ross 1977).

A range of more recent studies focusing on intergroup causal attribution, initially proposed and more recently reviewed by Hewstone (1989, 2012), suggest a more complex picture. These studies describe evidence of fundamental attribution error when making causal attributions for out-group members' behaviour if it is deemed positive or successful and that this effect is reversed for events that are considered negative or failures. They also found that self-serving bias extends beyond self to attributions of other in-group members' behaviour.

### 1.2 Attitudes towards automation

Attitudes towards automation also potentially affect how success and failure are attributed. Satellite navigation systems provide greater levels of navigational automation when compared with either web-based routing systems or traditional maps. Seppelt and Lee (2007) suggest that we have difficulty developing accurate knowledge of the capabilities and limitations of such highly automated systems. Discrepancies between actual and expected automation outcomes can reduce situational awareness, competency and reliance. Capturing users' general attitudes towards automation is complex and of little practical value. These attitudes are influenced by a wide range of contextual factors such as trust, personal experience, cost of failure and the fidelity of mental models of the automated system itself (Parasuraman and Manzey 2010). Therefore, there is merit in developing tools to examine attitudes towards specific technologies.

Safety science literature has a long history exploring the appropriate attribution of blame for negative events (Lowrance 1976), but this article focuses on attribution in the psychological sense rather than the determination of actual event triggers.

The following sections explore these issues in order to inform the design of navigational interfaces and the need to represent provenance and reliability information within navigation aids. This work also helps deepen our understanding of attribution and decision-making when working with geospatial data.

### 1.3 Eliciting attribution of navigation

An important factor to consider when studying attribution in the navigation domain is how to best elicit the nature of participants' attributions in given situations. Reporting personal experiences is the most naturalistic method and effective when participants have relevant recent experiences. However, self-report makes experimental manipulation difficult to achieve. Alternatively, abstract survey questions are relatively quick and easy to administer or manipulate, yet without the context of a real world scenario may lack ecological validity. The approach we use in this article, vignette-based methods, offers a compromise between the two as ‘a means of producing more valid and more reliable measures of respondent opinion than the “simpler” abstract questions more typical of opinion surveys’ (Alexander and Becker 1978, 1).

### 1.4 Hypotheses

Based on the review of literature as outlined earlier, we investigated the following hypotheses:

H1: Success of others' navigation affects attribution to external or internal factors.
H2: Failure of others' navigation affects attribution to external or internal factors.
H3: The attribution of others' success and failure when navigating is influenced by the type of navigation aid that they are using.
H4: Individuals' own success when navigating will tend to be attributed to internal factors.
H5: Individuals' own failure when navigating will tend to be attributed to external factors.
H6: Attribution of individuals' own success and failure when navigating is influenced by the type of navigation aid that they are using.

In order to test these hypotheses, external factors will be operationalised as attributions made to the navigation aid used, and internal factors as attribution made to personal navigation skill.

## 2. Method

An online survey was conducted exploring the attribution of navigation success and failures.

### 2.1 Participants

A total of 162 participants ranging in age from 18 to 60 years took part in the study. Of these participants, 49 were removed due to incomplete responses, thus the final analysis was performed on a total of 113 responses from 62 male and 51 female participants, falling into the same age range. When reporting experience with navigation aids the majority reported using satellite navigation and paper maps ‘more than once a month’ and web-based routing ‘more than once a week’. Of the final respondents, only 18 reported using one or more of these navigation aids less than once a month.

### 2.2 Design

#### 2.2.1 Section one: vignette study

The first section of the survey was a vignette study focusing on the attribution of others' navigation success and failure. This section had two independent variables: level of success and type of navigation aid used. Level of success and type of navigation aid used were described in three levels as illustrated in Table 1. Each participant took part in three of the nine conditions in a matched-pairs type 3 × 3 design. The combinations of navigation success and type of navigation were presented in a quasi-random fashion so that each participant was presented with each level of each of the independent variables once (i.e. each participant saw one and only one vignette in which the protagonist used a paper map, similarly they saw only one vignette in which the protagonist arrived on time).

**Table 1:** Independent variables.

Variable	Level	Text presented
Navigation success	On time	‘She/He takes the correct route and arrives on time.’
	Delayed	‘She/He takes a wrong turn on the way and arrives at the pub a little late.’
	Lost	‘After an hour of searching she/he is completely lost and has to ask a passer-by for directions.’
Type of navigation aid^a^	Satellite navigation	‘Following the directions from her/his satellite Navigation System (such as a TomTom or Garmin).’
	Routing engine	‘uses a web-based routing engine (such as Google Maps or Bing Maps)’
	Paper map	‘uses a paper map’

a Note that while the type of navigation aid was described, detailed information about integration with the aid, such as drawing on a map or printing the results from a routing engine, was not specified. It was thought that this level of specificity would increase the risk of participants commenting on an interaction with which they were not familiar, thus introducing a confounding variable.

**Figure 1:**
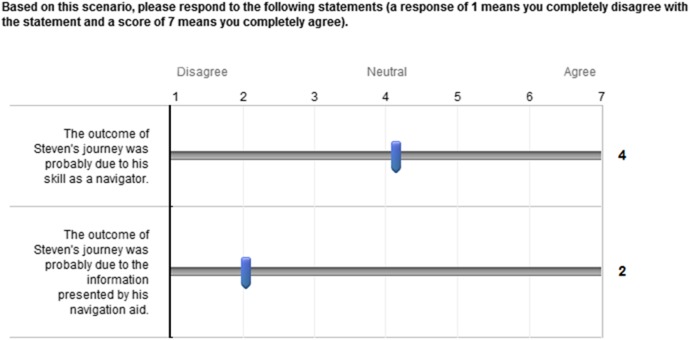
Questions presented with each vignette in section one of the survey.

The dependent measures were the attribution of navigation success to (1) the skill of the navigator and (2) the information provided by the navigation aid. Both were measured by a self-report on a seven-point interval scale question, as illustrated in Figure 1.

#### 2.2.2 Section two: personal experience

The second section of the survey invited participants to think of a memorable navigation experience they had undergone and to describe it in detail. They were then asked four specific questions about this navigation experience:

1. Which methods did you use to find your way? (List all that are appropriate):
2. Please describe how successful you feel your navigation was. Consider how long it took and any problems encountered along the way (wrong turns, having to alter your planned route etc.):
3. What do you think contributed to this level of success (your navigational ability, the quality of your navigation aids, luck, etc.)? Please give as much detail as possible:
4. Has the outcome of this navigation in any way influenced how you will navigate in the future? Please give details about how and why it will/will not influence your future behaviour.

### 2.3 Procedure

The entire procedure was completed online via Qualtrics,^1^ an online questionnaire suite. Participants were recruited though personal correspondence, social media and mailing lists to complete an online survey. The survey consisted of the two sections described earlier in addition to demographic questions, asking participants for their age group, gender and experience with various navigation aids. In total, the procedure took between 15 and 20 min to complete. Participants had the option to stop at any point in the procedure and return to the survey at a later date to complete it. Incomplete responses were discarded after 3 days of inactivity. Informed consent was obtained from each participant prior to his or her participation via an online briefing and consent form.

## 3. Results

This section describes the results of the qualitative and qualitative analysis of responses to the online survey.

### 3.1 Section one results

**Figure 2:**
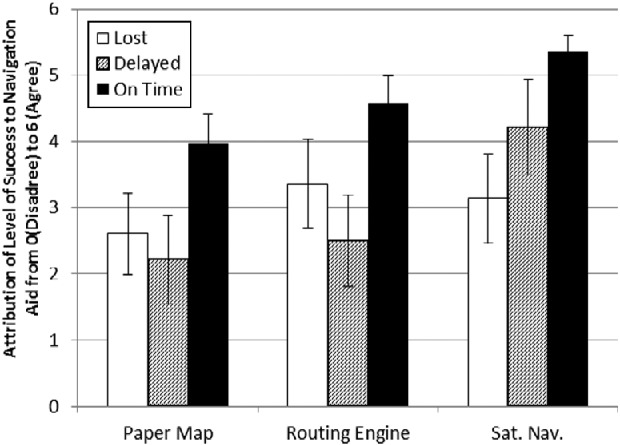
Mean scores and 95% confidence boundaries for ‘Navigation Aid Attribution’ in others.

**Figure 3:**
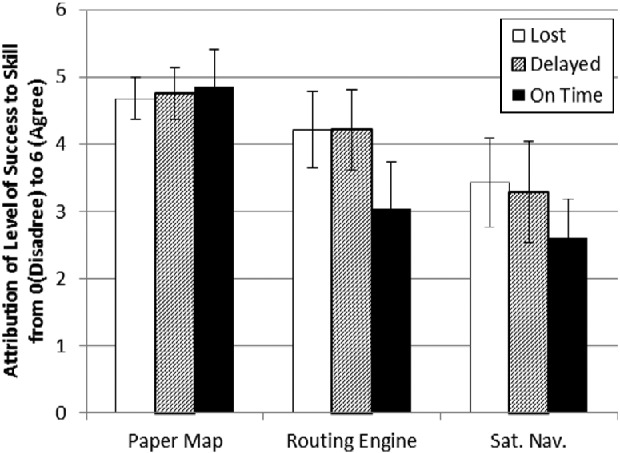
Mean scores and 95% confidence boundaries for ‘Skill Attribution’ in others.

Mean responses for both ‘Skill Attribution’ and ‘Navigation Aid Attribution’ were calculated for each set of Dependent Variables (see Figures 2 and 3). A MANOVA test was performed and was found to be significant at *p* ≤ 0.05 for ‘Level of Success’ (Wilks' λ = 0.230, df = 4) and ‘Type of Navigation Aid’ (Wilks' λ = 0.232, df = 4), but not the interaction between these effects (Wilks' λ = 0.527, df = 8, observed power = 0.711). Univariate testing with Greenhouse–Geisser sphericity correction revealed a number of significant effects at *p* ≤ 0.05, as shown in Table 2. Next, pairwise comparisons with Bonferroni correction were performed on both ‘Skill Attribution’ and ‘Navigation Aid Attribution’, in order to highlight the exact nature of the differences; again a number of significant effects were found at *p* ≤ 0.05 (see Table 3).

**Table 2:** Univariate MANOVA results for section one with Greenhouse–Geisser sphericity correction.

IV	Measure	Sum of squares	*F*	df
Level of success	Skill Attribution	19.865	3.840	1.807
	Aid Attribution	148.024	26.375	1.824
Type of navigation aid	Skill Attribution	115.722	21.926	1.736
	Aid Attribution	72.667	16.690	1.983

Note: All effects significant at *p* < 0.05.

**Table 3:** Pairwise comparisons for section one results.

Comparison by success	Level of Success 1	Level of Success 2	Mean difference	Standard error
Skill Attribution	Lost	Delayed	0.024	0.225
	Lost	On time	0.607	0.286
	**Delayed**	**On time**	**0.583**	**0.228**
Aid Attribution	Lost	Delayed	0.060	0.295
	**Lost**	**On time**	** − 1.595**	**0.244**
	**Delayed**	**On time**	** − 1.655**	**0.232**
Comparison by navigation type	Navigation Type 1	Navigation Type 2		
Skill Attribution	**Paper map**	**Routing engine**	**0.940**	**0.207**
	**Paper map**	**Sat. Nav.**	**1.655**	**0.246**
	Routing engine	Sat. Nav.	0.712	0.292
Aid Attribution	Paper map	Routing Engine	− 0.548	0.217
	**Paper map**	**Sat. Nav.**	** − 1.310**	**0.231**
	**Routing engine**	**Sat. Nav.**	** − 0.762**	**0.234**

Note: Significant results at *p* ≤ 0.05 after correction are highlighted in bold.

### 3.2 Section two results

The test-based nature of responses to section two meant that a combination of qualitative and quantitative analysis was required. Responses to question 1 (type of navigation aid used) were clustered into five categories: asking someone, satellite navigation, routing engine, paper map or multiple methods. Responses to questions two and three were less easily clustered; therefore a thematic analysis (Braun and Clarke 2006) was performed in order to identify groups of responses for further analysis. Five categories for ‘Level of Success’ and four for ‘Attribution of Success’ were revealed (see Table 4).

**Table 4:** Thematic analysis of section two responses for level of success and attribution of success.

Level of success theme	Number of comments	Example comments
Disastrous	9	‘It was a total disaster and meant instead of taking 1 hour to get there it took us 3 hours!’ – Participant 89‘Completely unsuccessful, I basically just ended up at my destination through pure luck’ – Participant 16
Annoying delays	13	‘Once I'd reached the rough area, it took me over an hour to find the hotel.’ – Participant 1‘Not very successful on the way there. Although we made it, and didn't take any wrong turns or have to backtrack, the journey was much longer than it should have been.’ – Participant 26
Mixed feelings	7	‘I found my way in the end although getting lost was frustrating. The upside was that using the map meant I got to know that area much better and the next time I had to drive out that direction I found my way without a map.’ – Participant 6‘The original navigation was not very successful, as I failed to find the station. However, the second attempt went well, and I had no problems once I had the more detailed information.’ – Participant 66
Positive experience	43	‘We only took one wrong turn on the way there which only delayed us for a minute as we were able to turn around swiftly. Apart from that we navigated successfully.’ – Participant 32‘Very successful – only one wrong turn, corrected almost instantly.’ – Participant 77
Complete success	16	‘The journey turned out to be excellent. I took no wrong turns and got to my destination with time to spare!’ – Participant 64‘No problems, did not take any wrong turns, followed planned route.’ – Participant 96
Attribution of success theme	Number of comments	Example comments
Egocentric view	36	‘I am a very experienced traveller and am rarely lost.’ – Participant 22‘My ability to match a description to a map, therefore I feel it was my ability to navigate.’ – Participant 81
Navigation aids	25	‘Google maps knowing about traffic/road closures etc. in real time.’ – Participant 34
External factors	9	‘Garmin definitely was the reason we got lost.’ – Participant 94‘It was partly bad luck … which led to us getting lost.’ – Participant 4‘Being able to ask people for directions.’ – Participant 12
Mixed	18	‘The quality of my navigation aids, my friend's ability to follow instructions while driving and bit of luck that my HSPA+ connection held out for the entire journey.’ – Participant 30‘The quality of the system and I usually have a good sense of direction.’ – Participant 93

In order to explore the interaction between the factors reported, a series of χ^2^ analyses were performed. For each of these, more than 20% of the cells had expected frequencies of less than five, so Yates' (1934) correction for small samples was applied.

The interaction between levels of success and success attribution was found to be significant at *p* ≤ 0.05 (χ^2^ = 38.140, df = 12) and subsequently a *post hoc* analysis of variation contributing to the χ^2^ results was performed (see Table 5 for details). These results support the hypotheses that successful navigation tends to be attributed to personal factors (H5) and failures to external factors (H6).

**Table 5:** χ^2^ residuals, after Yates' correction, for success level against success attribution.

	Egocentric view	Navigation aid	External factors	Mixed
Disastrous	** − 2.1**	**2.6**	1.1	− 1.6
Annoying delays	** − 2.5**	− 1.2	**4.9**	− 1.2
Mixed feelings	**2.8**	− 0.9	− 0.7	− 1.3
Positive experience	− 1.7	0.6	** − 3.1**	**4.2**
Complete success	**3.5**	− 1.1	** − 2.2**	− 0.2

Note: Significant residuals (absolute value ≥ 2.0) are highlighted in bold.

Similarly, the interaction between type of navigation aid used and success attribution was found to be significant (χ^2^ = 16.724, df = 12, significant at *p* ≤ 0.05) and subsequently a *post hoc* analysis was performed (see Table 6 for details). This finding supports our hypotheses that the type of navigation aid used influences attribution of navigation success (H4).

**Table 6:** χ^2^ residuals after Yates' correction for navigation aid against success attribution.

	Egocentric view	Navigation aid	External factors	Mixed
Asking someone	− 0.42	− 0.02	1.31	0.00
Satellite navigation	** − 4.29**	**3.07**	0.00	0.31
Routing engine	0.08	− 0.15	− 0.81	0.88
Map	**2.38**	− 0.84	− 0.34	− 0.19
Multiple methods	1.75	− 0.56	0.00	− 0.63

Note: Significant residuals (absolute value ≥ 2.0) are highlighted in bold.

Finally, each participant's reported navigation experience was content-analysed to ascertain whether it had an impact on their future navigations. This classification was then mapped separately against the success and attribution themes identified earlier. χ^2^ analysis was performed on both these sets of data but neither revealed significance at *p* ≤ 0.05 after Yates' correction for small samples (χ^2^ = 5.276 for Success and Impact, χ^2^ = 3.537 for Attribution and Impact).

### 3.3 Conclusions

Evidence was found to support each of our hypotheses at *p* ≤ 0.05:

H1 and H2 are supported by the MANOVA results and subsequent *post hoc* testing, revealing that others' navigations tend to be attributed to internal factors when unsuccessful and external factors when successful. These tests also support H3, showing that navigation using paper maps tends to be attributed to skill whereas navigation with a satellite navigation system tends to be attributed to the navigation aid itself.

H4 and H5 are supported by the χ^2^ results and *post hoc* testing of the interaction between ‘Level of Success’ and ‘Attribution of Success’ in section two of the questionnaire. These show a tendency to attribute delayed navigation to external factors and conversely completely successful navigations to personal factors.

The χ^2^ analysis exploring the interaction between type of navigation aid use and success attribution supports H6, revealing a tendency to attribute map-aided navigation to personal factors and satellite navigation-aided navigations to the aid itself.

## 4. Discussion

Our findings support previous research relating to the complex interaction between fundamental attribution error and self-serving bias as described by Hewstone (2012) and that these effects are a major influence on attribution of navigation success. In terms of the type of navigation aid across both sections of the study, success and failure in navigation tends to be attributed to skill when a map is used and the navigation aid itself when satellite navigation is used, with routing engines falling somewhere in-between.

Overall, our results suggest that more automated systems, such as satellite navigation, are perceived by individuals as having a highly influential role in outcome of events, regardless of whether the outcome is either success or failure. In addition, successes when navigating are unlikely to be remarkable or newsworthy. These phenomena, when combined with the difficulty that individuals have in understanding the capabilities and limitations of automated systems (Parasuraman and Riley 1997), may explain the media's tendency to focus on failures caused by satellite navigation systems (Simpson 2008).

These findings have implications when considering issues such as acceptance, trust and appropriation of navigation technologies. For example, if we consider technology acceptance models and derivations from them (Davis 1989; Venkatesh and Davis 2000), perceived usefulness is both strongly influenced by subjective experiences and in turn influences intention to use. Thus, misattribution of previous successes and failures can lead to distorted levels of perceived usefulness and ultimately inappropriate usage behaviour. This effect highlights the importance of supporting appropriate attribution though communication of the strengths and limitations of specific navigation solutions. Potential avenues for communication in this domain include highlighting the provenance of underlying data-sets, as explored by Idris, Jackson, and Abrahart (2011), and explicitly reporting uncertainty in automatic positioning and routing systems.

Future work in this area should observe the influences of skill and navigation aims upon the attributions made in individual navigation sessions in order to explore the accuracy of those attributions. A more in-depth exploration of concepts such as attributional styles and dimensions of attribution (Peterson et al. 1982) would also be valuable in unpacking the various individual differences and social factors at play. In general, there is a need for more research exploring the human factors behind the use of geospatial tools, such as user preference, technology acceptance and mental models.

## Footnotes

^1.^ http://www.qualtrics.com
